# Restoration of Bone after Trephining

**Published:** 1890-08-09

**Authors:** 


### SURGERY.

### RESTORATION OF BONE AFTER TREPHINING.

We gave some time since an account of a procedure by which the disc of bone was retained in vascular connection and replaced *in situ* after the operation. This, however, may not in every case be practicable, and some surgeons have tried to apply the principles on which skin grafting over widely denuded tracts, as after extensive ulceration of the leg, has been long successfully employed to the restoration of bone over the gaps (!) made in the cranium. Seydel took lamellæ from the tibia, but this method involved a serious wound in another part; and Jaksch has improved on the procedure by transplanting lamellæ of bone from the cranium of a young goose on to the granulating dura mater on the eighth day after the operation. Antiseptic dressings were applied, and on their removal ten days later all the eight lamellæ were found firmly adherent, and of a healthy, rosy colour. After another week the dividing lines had disappeared, and still a week later the whole surface had granulated. In the course of two months the bony covering was complete and strong. The skin, of course, was sacrificed, being replaced by cicatricial tissue. The other procedure, when feasible, has the advantage in this respect, that no disfigurement whatever remains.

